# Bioinformatic analysis of hub markers and immune cell infiltration characteristics of gastric cancer

**DOI:** 10.3389/fimmu.2023.1202529

**Published:** 2023-06-09

**Authors:** Chao Li, Tan Yang, Yu Yuan, Rou Wen, Huan Yu

**Affiliations:** ^1^ School of Pharmacy, Tianjin University of Traditional Chinese Medicine, Tianjin, China; ^2^ School of Pharmacy, Jiangxi University of Traditional Chinese Medicine, Nanchang, China

**Keywords:** gastric cancer (GC), hub markers, immune cell infiltration, WGCNA, LASSO

## Abstract

**Background:**

Gastric cancer (GC) is the fifth most common cancer and the second leading cause of cancer-related deaths worldwide. Due to the lack of specific markers, the early diagnosis of gastric cancer is very low, and most patients with gastric cancer are diagnosed at advanced stages. The aim of this study was to identify key biomarkers of GC and to elucidate GC-associated immune cell infiltration and related pathways.

**Methods:**

Gene microarray data associated with GC were downloaded from the Gene Expression Omnibus (GEO). Differentially expressed genes (DEGs) were analyzed using Gene Ontology (GO), Kyoto Gene and Genome Encyclopedia, Gene Set Enrichment Analysis (GSEA) and Protein−Protein Interaction (PPI) networks. Weighted gene coexpression network analysis (WGCNA) and the least absolute shrinkage and selection operator (LASSO) algorithm were used to identify pivotal genes for GC and to assess the diagnostic accuracy of GC hub markers using the subjects’ working characteristic curves. In addition, the infiltration levels of 28 immune cells in GC and their interrelationship with hub markers were analyzed using ssGSEA. And further validated by RT-qPCR.

**Results:**

A total of 133 DEGs were identified. The biological functions and signaling pathways closely associated with GC were inflammatory and immune processes. Nine expression modules were obtained by WGCNA, with the pink module having the highest correlation with GC; 13 crossover genes were obtained by combining DEGs. Subsequently, the LASSO algorithm and validation set verification analysis were used to finally identify three hub genes as potential biomarkers of GC. In the immune cell infiltration analysis, infiltration of activated CD4 T cell, macrophages, regulatory T cells and plasmacytoid dendritic cells was more significant in GC. The validation part demonstrated that three hub genes were expressed at lower levels in the gastric cancer cells.

**Conclusion:**

The use of WGCNA combined with the LASSO algorithm to identify hub biomarkers closely related to GC can help to elucidate the molecular mechanism of GC development and is important for finding new immunotherapeutic targets and disease prevention.

## 1 Introduction

GC is one of the most common malignancies in the human digestive tract. According to Global Cancer Statistics, GC has become the fifth most frequently diagnosed cancer and the third leading cause of cancer deaths, making it a major global health crisis (1). In China, the total number of new cases of GC in 2020 was 478,000, ranking 2nd in the number of incidences of malignant tumors and 373,000 deaths, ranking 3rd in the number of deaths from malignant tumors (2). The above figures are sufficient to show that GC is highly malignant, has a low survival rate and poor prognosis and is a serious threat to human health and life.

GC is a malignant disease caused by a combination of factors, such as *Helicobacter pylori* infection, unhealthy lifestyle, genetics and immune cell imbalance. The pathogenesis of GC is still not fully understood, but the activation of proto-oncogenes caused by the abovementioned oncogenic factors is an important molecular mechanism. The molecular mechanisms involved in the pathogenesis of the disease still need to be further elucidated. Clinical treatments for GC based on surgical resection, chemotherapy, radiotherapy or a combination of targeted therapies have difficulty completely removing the tumor lesions, and the tumor is prone to progression or recurrence with high toxic side effects, with a 5-year survival rate of patients as low as 10% to 15% (3–5). It is important to emphasize that GC is usually asymptomatic in the early stages, and some patients are already at an advanced stage when diagnosed, with a survival rate of only 24% (6). Therefore, it is important to develop effective biomarkers for the prognosis of gastric cancer and for targeted therapy.

The tumor microenvironment (TME), due to its key role in cancer progression and drug resistance, has emerged as a potential immunotherapeutic target for a variety of malignancies, including GC. The TME consists of different cell types, including immune and inflammatory cells (lymphocytes and macrophages), stromal cells (fibroblasts, adipocytes and pericytes), small cell organelles, RNA, blood vessels and lymphatic vessels, extracellular matrix (ECM) and secreted proteins. The cells involved in the GC immune microenvironment are called tumor infiltrating immune cells (TIICs) (7). Immunotherapy in the treatment of advanced GC improves survival and is associated with good survival in GC patients, according to the results of the CheckMate 649 case study presented at the European Society for Medical Oncology (ESMO) 2020 virtual meeting (8, 9). However, recent studies have found that abnormal activation of the immune system may also be a key factor in the development of GC (10). In short, tapping into immune cell-related targets is an effective pathway to optimize tumor immunotherapy.

Due to advances in genomic technology, bioinformatics analysis of gene expression profiles has become increasingly popular in molecular mechanistic studies and is playing an increasingly important role in the discovery of disease-specific biomarkers. Weighted gene coexpression network analysis was proposed by Zhang & Horvath in 2005 as a systematic algorithm widely used for bioinformatics data, avoiding the drawbacks of traditional differential gene screening methods, which tend to miss core molecules in the regulatory process and make it difficult to explore the whole biological system, and has been widely used to screen molecular diagnostic markers or therapeutic targets for complex diseases (11, 12). This provides a new way to predict the function of coexpressed genes and to find genes that play a key role in human disease. LASSO is a regression method that allows the calculation of correlation coefficients between variables and more accurate screening of variables (13). There have been a host of studies on screening GC biomarkers based on bioinformatics methods both domestically and internationally, but there are problems with a small sample size and a single data analysis method as well as lack of further experimental verification (14–16). Thus, this article comprehensively utilizes various bioinformatics methods to integrate and analyze gene datasets from multiple platforms, and expand sample size and validated by *in vitro* cellular experiments, for improving the scientific nature of bioinformatics analysis, and in order to more accurately explore the pathogenesis and therapeutic targets of GC, and provide molecular biology basis and new research ideas and directions for subsequent experimental research.

**Figure 1:**
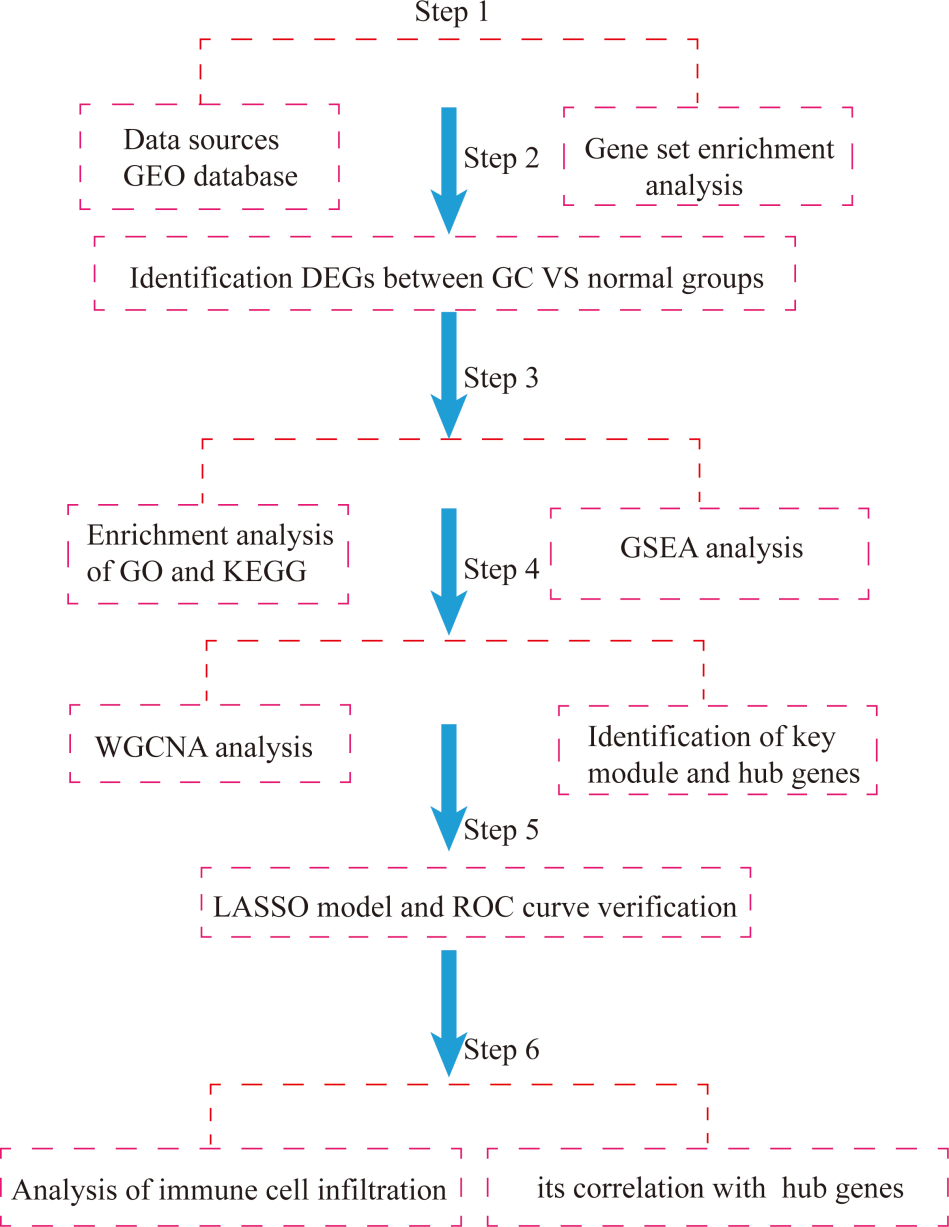
Flowchart of integrated bioinformatic analysis of hub markers and immune cell infiltration characteristics of GC.

Based on the above, this study used the GSE54129 and GSE65801 datasets to construct a gene weighted coexpression network by the WGCNA algorithm to screen out pivotal modules that are highly relevant to the development of GC, analyze the biological functions of the pivotal modules and use the LASSO regression model to screen key genes and validate them with the GSE118916 dataset, and then further identify important prognostic molecular markers and assess the extent of associated immune cell infiltration, with a view to providing new references for studying the development of GC, potential molecular mechanisms and therapeutic targets. Flowchart of our study was shown **Figure 1**.

## 2 Materials and methods

### 2.1 Expression data and clinical data collection

The flow chart of the study is shown in **Figure 1**. Acquisition of gene microarray data: Three gastric cancer datasets (GSE54129, GSE65801, GSE118916) were selected from the GEO database of NCBI (https://www.ncbi.nlm.nih.gov/geo/) based on the following three conditions: the samples were from human gastric tissue specimens, a case control group was available, and the number of samples was ≥20 to ensure the representativeness of the datasets. The datasets GSE79973, GSE65801 and GSE118916 were based on GPL570, GPL14550 and GPL15207, respectively. GSE54129 contained 111 cases of cancer and 21 cases of normal tissues; GSE65801 contained 32 cases of cancer and 32 cases of normal tissues; GSE118916 contained 15 cases of cancer and 15 cases of normal tissues. GSE118916 contained 15 cases of cancer and normal tissues. Detailed information is shown in **Table 1**.

**Table 1:** The main features of 3 selected datasets included in this analysis.

Database ID	Platform	Author	Year	Tissue sample	Number of treatment (GC group)	Number of control (normal group)
Training set GSE54129	GPL570	Liu B	2017	Gastric tissue	111	21
GSE65801	GPL14550	Hao L	2015	Gastric tissue	32	32
Validation set GSE118916	GPL15207	Li L	2019	Gastric tissue	15	15

### 2.2 Cells

The normal gastric cell line (GES-1) and gastric cancer cell line (MKN-45) were obtained from iCell Bioscience Inc., (Shanghai, China).

### 2.3 Data processing and analysis

The main analysis software used in this study was Rstudio desktop version, which is based on the Integrated Development Environment (IDE) for the R language, with better visualization, operability and simplicity. R packages are a collection of R language functions, example data and precompiled code. The main R packages used in this study are “WGCNA”, “clusterprofiler” and “ggpubr”.

#### 2.3.1 Data preprocessing

The downloaded raw data were preprocessed for information extraction, background correction and normalization, construction of gene expression matrices, and conversion of probe names to gene names, followed by the next step of analysis.

#### 2.3.2 Screening of differentially expressed genes (DEGs)

The R language (version 4.1.2) limma data package (Linear Models for Microarray Data) was used to normalize the data and screen for differentially expressed genes. |LogFC|>1 and corrected P<0.05 were used as conditions to screen for upregulated and downregulated genes. The pheatmap and ggplot packages in R language were used to plot heatmaps and volcano maps for DEGs, respectively.

#### 2.3.3 Construction of protein interaction networks

A protein interaction network (PPI) of differential genes was constructed using the String (http://string-db.org/) database, with an interaction score >0.4 as the threshold condition. The PPI network was imported into Cytoscape software for visualization, and the connectivity of the nodes was calculated. The systematic analysis of the interactions of a large number of proteins in biological systems is important for understanding the working principles of proteins in biological systems, the response mechanisms of biological signals and energy substance metabolism in specific physiological states such as diseases, as well as understanding the functional connections between proteins.

#### 2.3.4 Gene Ontology (GO) enrichment analysis of DEGs and Kyoto Encyclopedia of Genes and Genomes (KEGG) pathway analysis

GO analysis is a common method for enrichment studies of gene functions, which are classified into three categories: biological process (BP), molecular function (MF) and cellular component (CC). KEGG is a database that integrates a large amount of information on genomes, diseases, biological pathways and system functions. The GO function analysis and KEGG pathway analysis of differentially expressed genes were performed using the R 4.1.2 software clusterProfiler and enrichplot tools to derive the biological functions of DEGs, setting FDR *P*<0.05.

#### 2.3.5 Gene set enrichment analysis

Gene set enrichment analysis (GSEA) is a computational method in which all sequenced genes are first sorted in descending order of difference, and then the input gene set is ranked to determine its enrichment in different biological functions and signaling pathways.

GSEA is a computational method used to determine whether a set of *a priori* defined genes show statistically significant and consistent differences between two biological states. The downloaded GEO matrix files were collated and grouped into GC and normal groups. To verify the functional differences between the normal and GC groups in the dataset, we performed gene function enrichment analysis on the set of genes between the two groups using the gene set enrichment analysis (GSEA) method. The raw data were calculated by R language with corresponding P.adjust, q value, P value and log2 gene expression fold-change (FC). GSEA was performed using the cluster Profiler package, which is available on the Molecular Characterization Database website (https://www.gsea-msigdb.org/gsea/msigdb/index.jsp), to obtain the corresponding analysis. Pathways with |NES|>1, *P*<0.05 and FDR q<0.25 were generally considered to be significantly different.

#### 2.3.6 Construction of weighted gene coexpression networks

Genes with expression greater than all quartiles of variance were extracted and then imported into the R software platform “WGCNA” package to construct a GC-weighted gene coexpression network. Sample clustering trees were drawn, outlier samples were excluded, and sample numbers in the gene expression matrix were ensured to correspond to sample numbers in the clinical information. The optimal soft threshold β was calculated by the scale-free network, followed by the construction of the adjacency matrix by the power of the β operation. The topological overlap matrix (TOM) was then established to measure the similarity between genes, and the topological overlap matrix was used as the basic element to construct a hierarchical clustering tree. The dynamic hybrid cut method was used to divide and merge the modules and to draw the gene tree. After module partitioning, the module eigengene (ME) was calculated for each module and correlated with the clinical traits of GC patients and normal subjects, and the Pearson correlation coefficient was used to calculate the degree of correlation between the module eigenvectors and the clinical traits of the sample.

#### 2.3.7 Hub gene screening

To find the true core target genes, we took intersections of previously analyzed differential gene datasets and genes from the characterization module with the help of Venn plots. The relevant genes were then screened and used for further analysis.

#### 2.3.8 LASSO regression model building and ROC curve analysis

The LASSO regression model can calculate the correlation coefficients of the independent variables and incorporate the independent variables with coefficients that are not zero into the model, thus achieving dimensionality reduction. It can effectively avoid overfitting in dealing with high-dimensional data, multivariate covariance problems and overall variable selection and provides conditions for extracting characteristic genes. Receiver operating characteristic (ROC) curves are used to evaluate the accuracy of the model. After plotting the ROC curve, the area under the curve (AUC) value can be calculated, which is a probabilistic value that indicates the accuracy of the prediction model; the higher the AUC value, the better the model can classify the sample. In this study, the LASSO regression model was used to screen key genes that were highly correlated with the development of GC, and ROC curves were plotted to evaluate the accuracy of the LASSO regression model.

#### 2.3.9 Analysis of immune cell infiltration and its correlation with characteristic hub genes

Tumor-infiltrating immune cells were assessed using the ssGESA algorithm to estimate the proportion of immune cells in the tumor tissue. These immune cells included macrophage, central memory CD4 T cell, activated CD8 T cell, activated memory CD4 T cell, type 17 T helper cell, neutrophil and 28 other species. To improve accuracy, samples were screened at *P*< 0.05, and histograms of the proportion of each immune cell in all eligible samples, heatmaps of correlations between immune cells and violin plots of the proportion of immune cells in GC tissue versus normal tissue samples were plotted. Spearman correlation analysis was then used to analyze the association between hub genes and the 28 immune infiltrating cells, with correlation coefficients greater than 0 being positive and correlation coefficients less than 0 being negative, and the absolute value of the correlation coefficient representing strong, weak or no correlation, with *P ≤* 0.05 being considered statistically significant.

#### 2.3.10 Cell culture and RT-qPCR validation

Normal and cancer cells were cultured in RPMI-1640 medium (Gibco) at 37 °C with 5% CO2, and 10% fetal bovine serum (Gibco) and 1% penicillin-streptomycin solution (Gibco) were added to all media, and the cells could be processed for passaging when they were logarithmically grown.

Total RNA was extracted from normal gastric cells (GES-1) and gastric cancer cells (MKN-45) using TRIzol. Real-time fluorescence quantitative PCR was performed using HiScript^®^ II Q RT SuperMix kit and SYBR Green Master Mix (Vazyme, Nanjing, China). Data were normalized to the GAPDH expression level of the internal reference control, and the relative expression levels of hub genes in different groups were calculated using the 2^-ΔΔCt^ method. The primers were synthesized and designed by wuhan huayan Biotechnology CO., LTD (Wuhan, China). The primer sequences are shown in **Table 2**.

**Table 2:** RT-qPCR primer sequences.

Gene	Primer	Sequence (5’-3’)	PCR Products
Homo GAPDH	Forward	TCAAGAAGGTGGTGAAGCAGG	115bp
	Reverse	TCAAAGGTGGAGGAGTGGGT	
Homo ADH7	Forward	GATGGCACCACCAGATTTACA	282bp
	Reverse	CCTAGATGCACCAGCTGACTTA	
Homo CWH43	Forward	CCCAGGAGGTGTCTACGCT	241bp
	Reverse	CAGTTTTCTCTCATAGGCTTTA	
Homo SCNN1B	Forward	GGAGCGGGACCAAAGCA	125bp
	Reverse	GCAGCCAGACGATGTTA	

#### 2.3.11 Statistical analysis

Analysis of variance results were obtained by R software (version 4.2.3), and t-test was used for comparison between the two groups, with *P*<0.05 being a significant difference.

## 3 Results

### 3.1 Screening of DEGs

**Figure 2:**
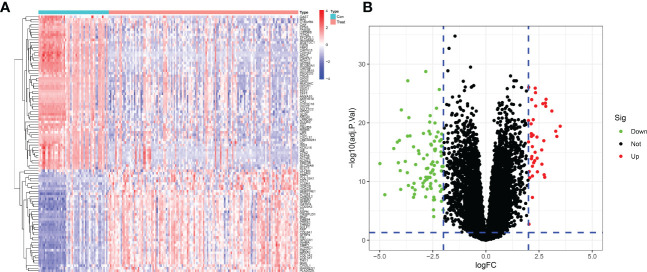
Differentially expressed genes between GC patients and healthy controls. **(A)** Heatmap of the top 50 up- and down-regulated genes. **(B)** DEGs volcano plot between healthy controls and GC tissue.

After merging and eliminating the batch effect of the GSE54129 and GSE65801 datasets, 133 differentially expressed genes were screened to obtain a heatmap and volcano map using differential genes. In this paper, the differentially expressed genes were analyzed by hierarchical clustering using the”pheatmap” package in R. The top 50 differentially expressed genes heatmap was output, with red representing increasing gene expression levels and green representing decreasing gene expression levels. Differential gene expression profiles existed between the normal control and GC groups (**Figure 2A**). The volcano plot (**Figure 2B**) can reflect the overall gene expression, the horizontal coordinate represents -log10 (corrected *P* value), the vertical coordinate represents log (fold change), each point represents a gene, red points represent differential gene expression upregulation, green points represent differential gene expression downregulation, and black points represent differentially expressed genes that are not significant.

### 3.2 Results of functional enrichment analysis of DEGs and their PPI construction

**Figure 3:**
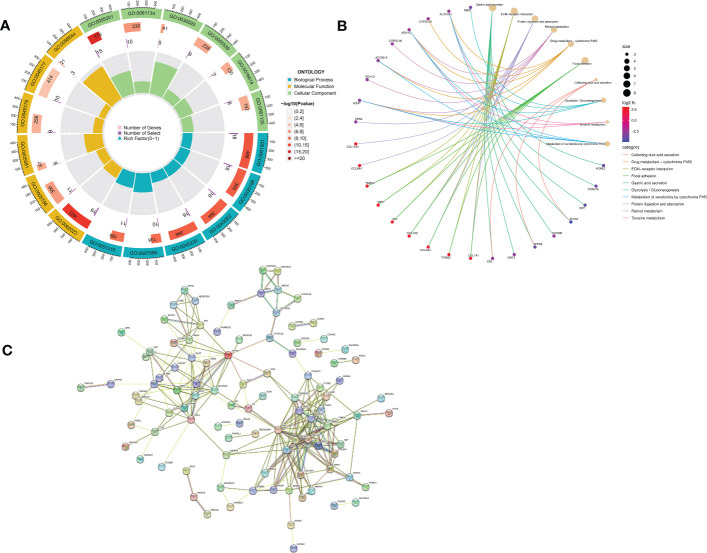
Functional enrichment analysis of DEGs and their PPI construction. **(A)** GO enrichment analysis. The first circle indicates the name of the GO; the second circle represents the number of genes on each GO. (The redder the color, the more significant the enrichment of DEGs); the third circle indicates the number of differential genes enriched on each GO term; and the fourth circle represents the proportion of genes. **(B)** KEGG pathway enrichment analysis. The different line colors indicate the different pathways to which they belong. Yellow dots are pathways, with larger dots indicating more genes involved. The other dots represent genes, the redder the gene the higher the expression level in GC patients and vice versa, the bluer the color. The top eight pathways for significant enrichment of differential genes were demonstrated. **(C)** Protein-protein interaction (PPI) network.

**Figure 4:**
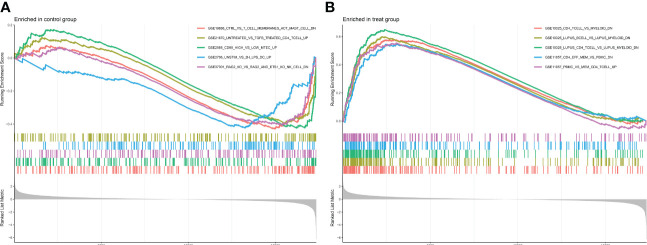
Enrichment plot for GSEA. **(A)** Active gene sets in healthy controls. **(B)** Active gene set in GC group.

GO enrichment analysis of 133 differential genes was performed using the clusterProfiler package in R. The differential genes were normalized in terms of biological pathways involved, function and cellular localization (**Table 2**). The GO analysis showed that these genes were mainly involved in the following biological processes: extracellular matrix organization, extracellular structure organization, external encapsulating structure organization and digestion. The main MF categories included extracellular matrix structural constituent, peptidase regulator activity, extracellular matrix structural constituent conferring tensile strength, and glycosaminoglycan binding. The main CCs were collagen-containing extracellular matrix, endoplasmic reticulum lumen, collagen trimer and basal cells (**Figure 3A**). KEGG pathway analysis revealed that these genes were mainly enriched in gastric acid secretion, ECM-receptor interaction, protein digestion and absorption and amino acid metabolism (**Figure 3B**). To further understand the potential connections between the proteins, we constructed a PPI network of DEGs with a PPI enrichment *P* value of <1.0e-16. The network consisted of 263 edges and 131 nodes with tight connections between nodes (**Figure 3C**). Furthermore, GSEA showed that the gene set was mainly enriched in the normal group of macrophages, B cells, CD4 T cell, T cell, cytokines and immune organs (**Figures 4A, B**), and the top 5 significantly enriched gene sets in normal control group and GC group see **Table 3** for details.

**Table 3:** Top 5 significantly enriched gene sets in normal control group and GC group.

Gene set name	NES	*p*value	*p*.adjust	qvalues
Enriched in normal control group				
GSE19888 CTRL VS T Cell membranes ACT mast Cell down	-1.692957907	0.000161482	0.001895759	0.001302505
GSE21670 Untreated vs TGFB treated CD4 T Cell up	-1.554264308	0.001274963	0.009257259	0.006360315
GSE2585 CD80 high vs low MTEC up	-1.657193329	0.000254598	0.002690672	0.00184866
GSE2706 Unstim VS 2H LPS DC up	-1.634072618	0.000987185	0.007658545	0.005261899
GSE37301 Rag2 KO VS Rag2 and Ets1 KO NK cell down	-1.593873519	0.000661997	0.005698322	0.003915103
Enriched in treat (GC) group				
GSE10325_CD4 T Cell VS Myeloid down	2.298424482	1.00E-10	1.08E-08	7.44E-09
GSE10325 Lupus B Cell VS Lupus Myeloid down	2.403164632	1.00E-10	1.08E-08	7.44E-09
GSE10325 Lupus CD4 T Cell VS Lupus Myeloid down	2.593696725	1.00E-10	1.08E-08	7.44E-09
GSE11057 CD4 Eff Mem VS Pbmc down	2.176921036	1.00E-10	1.08E-08	7.44E-09
GSE11057 Pbmc VS Mem CD4 T Cell up	2.206216446	1.00E-10	1.08E-08	7.44E-09

### 3.3 Identification of key modules based on WGCNA

**Figure 5:**
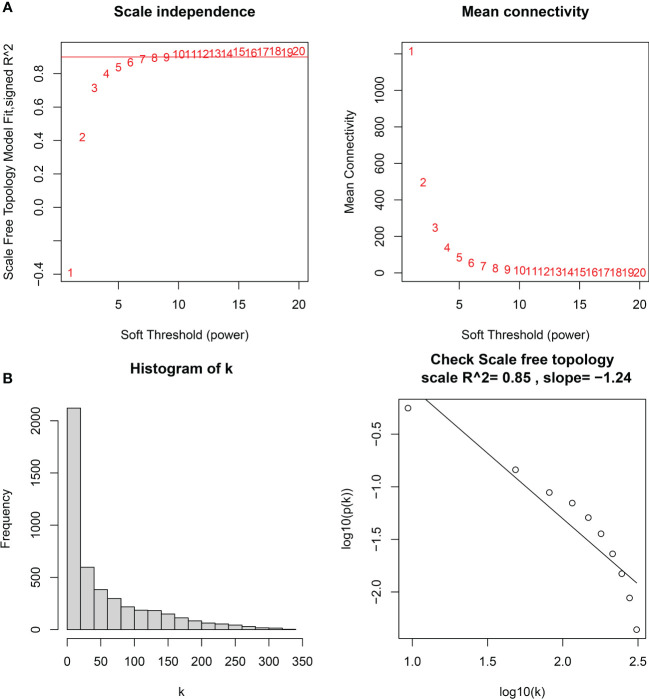
**(A)** Soft thresholds for determining the best scale-free topological model fit index (left) and average connectivity (right), with the red horizontal line indicating R^2^ = 0.9. **(B)** The distribution of the connectivity of each node in the network (left) and node degree power distribution (right).

**Figure 6:**
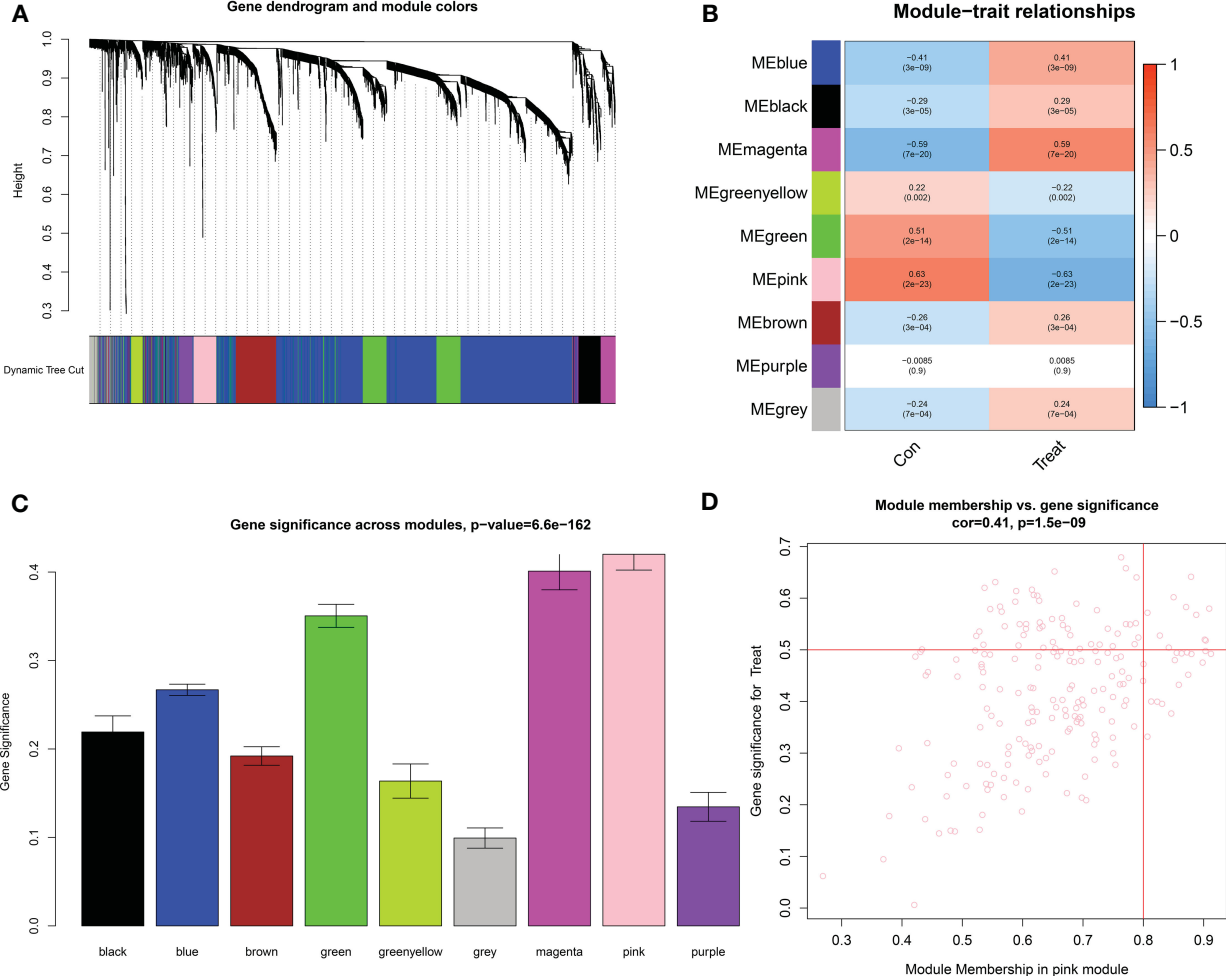
Identification of key modules based on WGCNA. **(A)** GC-related gene clustering dendrogram. In the figure, the top half is a hierarchical clustering tree diagram of the genes, and the bottom half is the gene modules, or network modules. Genes with relative relatedness are located on the same or adjacent branches. **(B)** Heatmap of correlation analysis of the modules and clinical traits. **(C)** Gene significance in the modules. **(D)** Scatter plots of GS score and MM for genes in the pink module.

The downloaded dataset was first preprocessed, and samples were screened to remove missing values to ensure reliable network construction, yielding 196 samples and 17,348 genes for subsequent analysis in the construction of WGCNA. A hierarchical clustering tree was created based on dynamic hybrid cuts using scale-free coexpression networks and topological overlap. Based on the scale-free topology criterion, the optimal soft threshold β = 6 was determined based on the scale-free fit index R^2 = ^0.9. A total of nine modules were obtained by dynamic hybrid cutting (**Figures 5A,  B**), corresponding to the colors black, blue, brown, green, green-yellow, gray, magenta, pink and purple, and the numbers of module genes were 223, 2574, 446, 614, 101, 115, 159, 201 and 125, in that order. The most relevant hub modules to GC were screened by calculating the correlation coefficient (R) and *P* value for each module (**Figure 6A**). The heatmap from this study shows that the pink module (201 genes) was highly positively correlated with GC (R = 0.63, *P* = 2e-23) (**Figures 6B, C**), and subsequently, the 201 core genes of the pink module (cor = 0.41, *P* = 1.5e-09) were screened for subsequent analysis based on GS > 0.5 and MM > 0.8 (**Figure 6D**).

### 3.4 Screening for hub genes

**Figure 7:**
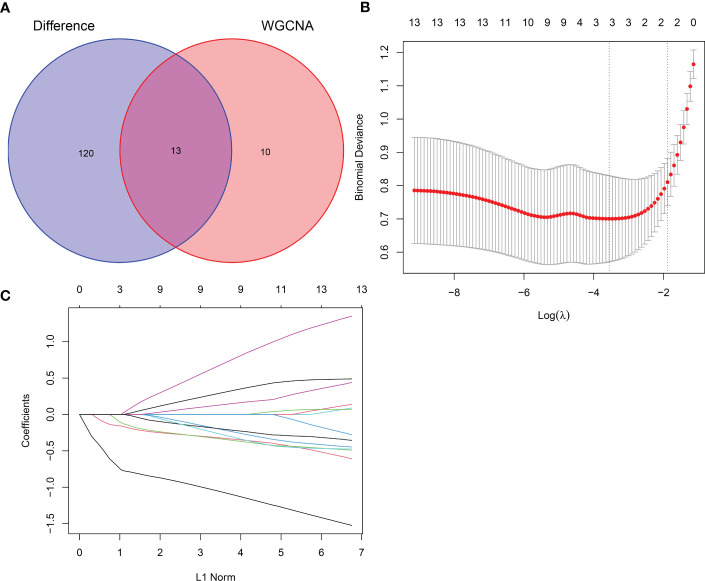
LASSO screening for hub genes. **(A)** Venn diagram of intersecting genes between DEGs and the pink module. **(B)** Coefficients distribution trend of LASSO regression. **(C)** Distribution of hub genes in cross validation.

Thirteen crossover genes were obtained after taking the intersection of the DEG dataset and the gene set in the feature module (**Figure 7A**). Subsequently, LASSO analysis was used to screen three genes from the crossover genes as pivotal genes for GC, including ADH7, CWH43 and SCNN1B (**Figures 7B, C**).

### 3.5 Identification and validation of differential expression analysis of key genes and their diagnostic value

**Figure 8:**
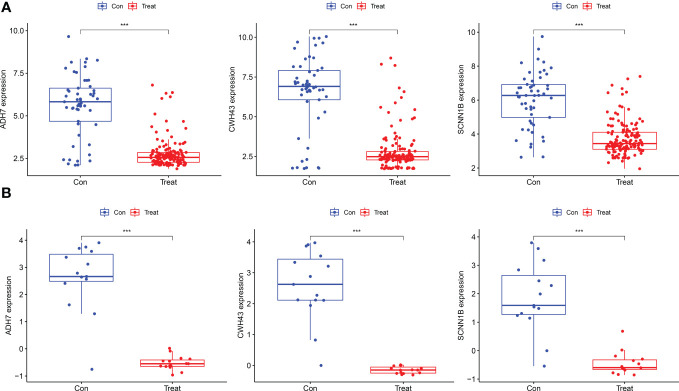
Expression levels of the three Hub genes between the normal control and GC groups. **(A)** Boxplot of these hub genes in the training dataset. **(B)** Boxplot of hub genes in the validation dataset. (***P<0.001).

**Figure 9:**
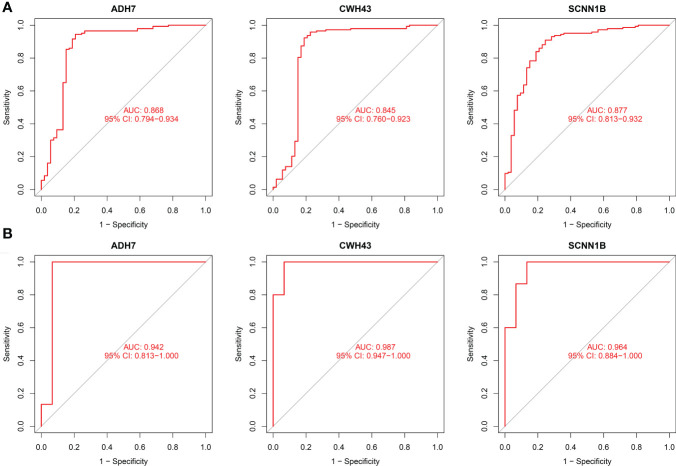
Diagnostic value of the three genes. **(A)** ROC curves of hub genes in the training dataset. **(B)** ROC curves of hub genes in the validation dataset.

The screened hub genes were extracted for expression to construct differential expression box plots. The differential expression box plot showed that all three key genes were underexpressed in GC patients (*P* < 0.001) (**Figure 8A**). The AUC areas for the three gene models were 0.868, 0.845 and 0.877, respectively (**Figure 9A**), indicating that the model is highly accurate and that ADH7, CWH43 and SCNN1B may be involved in affecting the development of GC. Subsequently, the independent dataset GSE118916 was used as the validation dataset to identify their expression levels and diagnostic value to further validate the clinical application of the pivotal genes. The results showed that the expression levels of ADH7, CWH43 and SCNN1B in the GC group were significantly lower than those in healthy controls in the validation set (*P* < 0.001), which was consistent with the results of the training set data (**Figure 8B**). ROC curves were used to further validate the diagnostic value of the three pivotal genes in the validation dataset. The results showed that ADH7, CWH43 and SCNN1B had high diagnostic value with AUC values of 0.942, 0.987 and 0.964, respectively (**Figure 9B**).

### 3.6 Analysis of immune cell infiltration and its correlation with characteristic hub genes

**Figure 10:**
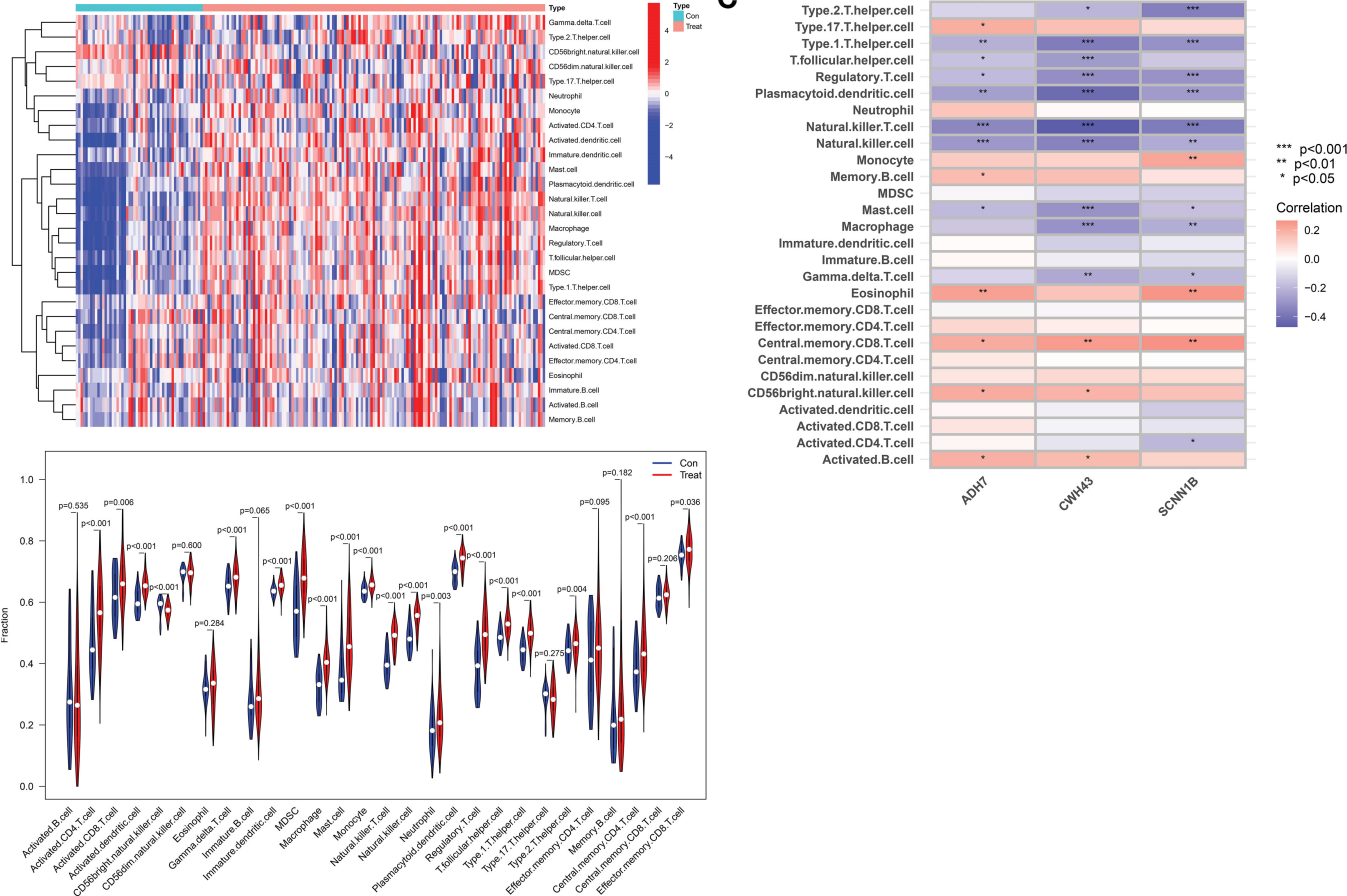
Analysis of immune cell infiltration and its correlation with characteristic hub genes. **(A)** Heat map of immune cell infiltration between normal control and GC group **(B)** Violin diagram of the difference in immune cell infiltration between normal controls and GC. **(C)** Analysis of the association of 3 Hub genes with immune cells.

Immune cell infiltration was assessed by the ssGSEA algorithm on tissue samples from the dataset, involving a total of 28 immune cell species. The majority of immune cells were found to be highly infiltrated in GC tissue (**Figure 10A**). Among them, activated CD4 T cell, activated dendritic cell, CD56 bright natural killer cell, γδ T cell, immature dendritic cell, MDSC, macrophage, mast cell, monocyte, natural killer T cell, natural killer cell, plasmacytoid dendritic cell, regulatory T cell, T follicular helper cell, type 1 helper cell, central memory CD4 T cell and regulatory T cell were extremely significantly increased in GC tissues (*P*<0.001), and activated CD8 T cell (*P*=0.006), neutrophil (*P*=0.003), type 2 helper cell (*P*=0.004) and e 0.004) and effector memory CD8 T cell (*P*=0.036) were also significantly increased in GC tissue. In contrast, activated B cell (*P*=0.535), CD56bright natural killer cell (*P*=0.600), eosinophil (*P*=0.284), immature B cell (*P*=0.065), type 17 T helper cell (*P*=0.275), effector memory CD4 T cell (*P*=0.095), memory B cell (*P*=0.182) and central memory CD8 T cell (*P*=0.535) did not differ significantly in GC tissue (**Figure 10B**). We then performed a correlation analysis to further explore the association of the hub genes with the 28 immune cells. We found that ADH7, CWH43 and SCNN1B were significantly associated with type 1 helper cell, T follicular helper cell, regulatory T cell, plasmacytoid dendritic cell, natural killer T cell, and natural cells. In addition, CWH43 and SCNN1B were also negatively correlated with type 1 helper cell, macrophages and γδ T cell (*P*<0.05). Interestingly, SCNN1B was also negatively correlated with activated CD4 T cell (*P*<0.001, *P*<0.01, *P*<0.05). ADH7 and CWH43 were significantly positively correlated with CD56 bright natural killer cell (*P*<0.05), while SCNN1B was significantly positively correlated with monocyte (*P*<0.01) (**Figure 10C**). These results suggest that hub genes may influence malignant tumor progression by regulating the abundance of infiltrating immune cells in the nodal GC tumor microenvironment.

### 3.7 Expression of hub genes in two groups of cells

**Figure 11:**
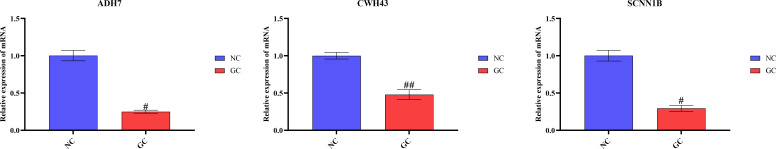
RT-qPCR validation of hub gene mRNA in different groups. The data presented are means ± SD (n=3). ^#^ *P <*0.05 and ^##^ *P <*0.01 relative to the control group.

To verify our predicted results, we did further validation by *in vitro* cellular experiments. As shown in **Figure 11**, it was confirmed that ADH7, CWH43 and SCNN1B all showed low expression in gastric cancer cells (*p* < 0.05). This is consistent with the results of our bioinformatics analysis.

## 4 Discussion

In recent years, the understanding of the pathogenesis of gastric cancer has been deepened, and a series of targeted drugs have been explored continuously, but the current exploration of gastric cancer targets is not comprehensive and in-depth enough for a multitarget, multilevel systemic therapy (17). Therefore, it is of great clinical importance to expand the research and discovery of potential targets for gastric cancer. Based on the multilevel concept of “disease-phenotype-molecule”, combined with the application and development of computer technology and artificial intelligence in the field of medical biology, bioinformatics has become one of the necessary tools for molecular marker research based on big data, which can be used to screen molecular markers related to disease phenotypes (18, 19). Individualized treatment and predictable outcomes of molecular pathways associated with gastric cancer have opened up many research directions, such as the use of molecular markers as useful tools in clinical work to assist in the diagnosis and treatment of gastric cancer patients, to assess the efficacy of treatments and to explore new therapeutic modalities (20, 21).

In this study, we obtained gastric cancer and normal tissue gene microarray datasets from the GEO database and performed DEG analysis on these combined datasets. GO and KEGG analyses showed that gastric cancer tissues differed significantly from normal tissue cells in BP, CC, and MF, mainly in biological processes such as collagen catabolic processes, extracellular matrix disassembly, and collagen protofibril tissue synthesis. The differential cellular components included extracellular regions, protein extracellular matrix, collagen trimer, etc., and both BP and CC play an important role in the migration of tumor cells. The extracellular matrix (ECM) is a loose connective tissue located outside the cell and contains a variety of biomolecules, such as collagen, adhesion factors, glycoproteins, and cytokines (22). It is physiologically important in intercellular signaling, intercellular interactions and regulation of cell proliferation, differentiation and migration (23). The ECM has been shown to be an independent risk factor for lymph node metastasis in early gastric cancer. Furthermore, the overall results of KEGG enrichment suggest that GC is accompanied by disturbed gastric acid secretion, amino acid metabolism and energy metabolism. The answer to this phenotype is well documented in the previous literature. Tumor cells are able to survive and proliferate in a nutrient-poor microenvironment through metabolic reprogramming, where abnormal glucose metabolism plays an important role in maintaining the malignant character of the tumor (24). Tumor cells obtain the energy necessary for growth and proliferation by glycolysis, even in conditions of adequate oxygen (25). Excessive gastric acid promotes the progression of gastric cancer. Gastrin, an inducer of gastric acid secretion, has been shown to be a valuable screening marker for gastric cancer (26, 27).

Most studies are currently based only on systems biology methods or machine learning algorithms for cancer marker screening. The use of a single systems biology approach or machine learning algorithm for data analysis may lead to some missing data or too much confounding data, so the combination of two or more methods can improve the confidence in the results (28). In this study, three biomarkers, ADH7, CWH43 and SCNN1B, were included in the model that used multiple bioinformatics methods to screen for gastric cancer. Based on the literature available to date, ADH7 belongs to the alcohol dehydrogenase family, a gene expressed mainly in the upper gastrointestinal tract, and has been shown to be involved in the metabolism of xenobiotics by cytochrome P450: it is associated with the metabolism of ethanol that occurs in gastroesophageal tissues and is then absorbed into the bloodstream. In addition, single nucleotide polymorphisms in ADH7 are susceptibility factors for cancer and drug dependence (29). SCNN1B encodes the β subunit of the epithelial sodium channel (ENaC), which is involved in the control of transepithelial transport of water and electrolytes and cell differentiation in different organs. Current studies on ENaC in cancer have shown that in breast cancer and neuroblastoma, SCNN1A gene silencing caused by hypermethylation in the promoter region of the SCNN1A gene, which encodes the α subunit of ENaC, is the main reason for the poor prognosis of patients with these tumors and diseases. Recently, SCNN1B was found to inhibit the growth and metastasis of gastric cancer cells, and the expression level of SCNN1B was positively correlated with the survival rate of gastric cancer patients and reduce the expression level of Glucose-Regulated Protein 78 [GRP78, Recent studies have also found that GRP78 expression is elevated in cancer cells and plays an important role in the development of cancer tumors (30, 31)]. In addition, activation of downstream proteins leads to caspase-dependent apoptosis and cell cycle arrest through induction of the unfolded protein response (UPR) (32–34). A recent study identified CWH43 as a prognosis-related gene in colorectal cancer (CRC), but little is known about its function (35).

The GC tumor microenvironment is highly complex and heterogeneous, tumor-associated immune cells play a role in tumorigenesis, development, invasion and metastasis, and the type and proportion of their infiltration are closely related to the clinical outcome of patients (36, 37). Therefore, the investigation of immune cell infiltration and its correlation with characteristic hub genes is also important for the pathogenesis, prevention and treatment of GC. In this study, we used ssGSEA to assess the expression levels and dynamic regulatory processes of 28 immune cell types in GC. The results showed significant differences in the pattern of immune cell infiltration between normal gastric and GC tissues, which to some extent indicated an imbalance in the immune response in GC. Tumor-associated macrophages (TAMs) are important components of the gastric cancer tumor microenvironment, which can influence the malignant biological behavior of gastric cancer and play a key role in gastric carcinogenesis and metastasis (38, 39). In the tumor microenvironment, TAMs secrete a large number of inflammatory factors, growth factors, chemokines and proteases through crosstalk with gastric cancer cells and various other cells, which play an active role in tumor growth, inhibition of apoptosis, angiogenesis and lymphatic metastasis (40, 41). In addition, myeloid inhibitory cells (MDSCs) are diverse bone marrow progenitor cells that produce arginase 1 (ARG1) to promote tumor cell growth and suppress immune cell function (42). CD4 T cells can be differentiated into four main subpopulations: Th1 cells, Th2 cells, regulatory cells (Tregs) and Th17 cells. The imbalance in the ratio of T lymphocytes alters the immune microenvironment of tumors, thus facilitating the proliferation, invasion and metastasis of tumor cells. Immunosuppressive effector cells modulate the intensity of the body’s immune response, attenuate immune damage, and mediate immune escape by suppressing the antitumor immune response, thereby promoting tumor progression. Previous studies have shown that a large number of immune cells and inflammatory factors are present in the tumor microenvironment of GC, and the number and phenotype of immune cell subpopulations in GC tissues are closely related to the development of GC and the prognosis of patients (43–45). To further reveal the potential mechanism of the differential expression of hub genes on the predictive value of the immune microenvironment in GC, this study analyzed these markers with infiltrating immune cells and found that the expression of these three biomarkers was significantly and negatively correlated with the level of immune infiltration of immune cells that were significantly upregulated in GC. This suggests that these genes may influence the progression of GC by affecting the level of immune infiltration as well as the interactions between immune cells. In short, these correlations may reveal potential molecular mechanisms underlying GC development and suggest that ADH7, CWH43 and SCNN1B play important roles in the GC immune microenvironment.

Although there are potential suggestions from this study for the early detection of gastric cancer and the corresponding treatment, there are still some limitations to consider. First, the sample size used in this trial may limit the generalizability of the study findings, and therefore, further evaluation in a larger cohort and in a different population would provide stronger evidence. Second, this study primarily utilized retrospective transcriptome analysis data and lacked validation. Therefore, *in vitro*, *in vivo* and prospective data still need to be collected to validate the real-world clinical significance of the identified DEGs and core genes in relation to gastric carcinogenesis, progression and prognosis. Finally, more experiments are needed to elucidate the upstream regulatory pathways and downstream mechanisms of the identified key differentially expressed genes.

In conclusion, the present study screened and validated the key genes ADH7, CWH43 and SCNN1B, which are significantly associated with GC development, based on the GEO public database, through a combination of WGCNA and lasso regression models, providing a molecular basis for the early diagnosis and treatment of GC, as well as for immunotherapy research and the development of new targeted drugs.

## Data availability statement

Publicly available datasets were analyzed in this study. This data can be found here: https://www.ncbi.nlm.nih.gov/geo/query/acc.cgi?acc=GSE54129, https://www.ncbi.nlm.nih.gov/geo/query/acc.cgi?acc=GSE65801, https://www.ncbi.nlm.nih.gov/geo/query/acc.cgi?acc=GSE118916.

## Author contributions

CL: Conceptualization, Methodology, Software, Writing-Original Draft. TY: Methodology, Validation, Writing-Original Draft. YY: Visualization, Investigation. RW: Formal analysis, Software. HY: Writing-Review & Editing, Supervision, Funding acquisition. All authors contributed to the article and approved the submitted version.
